# Mixed *Streptococcus pneumoniae* and *Streptococcus pyogenes* meningitis in an immunocompromised adult patient: a case report

**DOI:** 10.1186/s13256-015-0763-9

**Published:** 2015-11-29

**Authors:** Clémence Demerle, Vadim Ivanov, Cédric Mercier, Régis Costello, Michel Drancourt

**Affiliations:** 1 Aix Marseille Université URMITE, UMR CNRS 7278, IRD 198, INSERM 1095, Faculté de Médecine, 27, Boulevard Jean Moulin, 13005 Marseille cedex 5, France; 2 Service d’Hématologie et Thérapie Cellulaire, Centre Hospitalier Universitaire La Conception, 147 Boulevard Baille, 13005 Marseille cedex 5, France; 3 Unité de recherche sur les maladies infectieuses et tropicales émergentes, Faculté de Médecine, 27 Boulevard Jean Moulin, 13385 Marseille cedex 5, France

**Keywords:** Meningitis, Purulent otitis, *Streptococcus pneumoniae*, *Streptococcus pyogenes*

## Abstract

**Introduction:**

Community-acquired meningitis is a monomicrobial infection caused by either viruses or bacteria in the vast majority of patients. We report here one exceptional case of a patient with mixed bacterial meningitis due to *Streptococcus pneumoniae* and *Streptococcus pyogenes*.

**Case presentation:**

We report the case of a 68-year-old immunocompromised Caucasian man suffering from otitis and then meningitis caused by *Streptococcus pneumoniae* and *Streptococcus pyogenes*. Bacteria were undistinguishable by direct microscopic examination of the cerebrospinal fluid. He responded well to treatment with cefotaxime and dexamethasone, with no sequelae observed at the 4-month follow-up.

**Conclusions:**

This first reported case of mixed *S. pneumoniae* and *S. pyogenes* meningitis illustrates the life-threatening consequences of barotrauma in immunocompromised patients suffering from otorhinolaryngeal infections.

## Introduction

Community-acquired meningitis is a monomicrobial infection caused by only one pathogen, either a virus or a bacterium, in the vast majority of patients. Among bacterial pathogens responsible for meningitis, the prevalence of *Streptococcus pneumoniae* is declining, although this organism is still responsible for more than 50 % of bacterial meningitis cases [1]. It is diagnosed by real-time polymerase chain reaction (PCR)-based detection of specific DNA sequences in the cerebrospinal fluid (CSF), yet direct microscopic examination of the CSF after Gram staining is routinely used as a screening method to establish a presumptive etiology [2]. Part of the relative specificity of Gram staining is based on the common fact that only one bacterium should be detected in the CSF.

We report here one exceptional case of a patient with mixed bacterial meningitis due to *S. pneumoniae* and *Streptococcus pyogenes* where routine Gram staining was not reliable for the final diagnosis.

## Case presentation

A 68-year-old Caucasian man was admitted 1 day after flying back from Madagascar to Marseille, France. He was in remission from chronic lymphoid leukemia with no chemotherapy for 6 months and had had unilateral purulent otorrhea for several days. At the time of admission, our patient’s temperature was 38 °C and he presented with a stiff neck and confusion. His left ear was painful and inflamed, and an examination revealed pus without tympanic membrane perforation. A cranial computed tomography (CT) scan showed evidence of a left mastoid infection without bone erosion, cholesteatoma or brain abscess. Ear pus was sampled by Sigma-Transwab (Elitech France, Puteaux, France). CSF was collected after a lumbar puncture and our patient received 300 mg/kg cefotaxime and 20 mg dexamethasone [3, 4]. Relevant biological parameters included pancytopenia with 3.68 T/L red cells, a hemoglobin level of 117 g/L, and 1.13 G/L leukocytes including 0.39 G/L lymphocytes and 74 G/L platelets. Appropriate point-of-care (POC) tests excluded malaria, dengue and Chikungunya viral infections [5].

Direct microscopic examination of the Gram-stained CSF revealed 930 polymorphonuclear cells and 170 red cells per cubic millimeter, along with numerous Gram-positive cocci. Our patient’s CSF contained 3.63 g/L total protein and 1.31 mmol/L glucose. *S. pneumoniae* antigen detection was positive (BinaxNOW, Alere, Jouy-en-Josas, France) [6] along with positive real-time PCR detection of *S. pneumoniae* Lyt-A and *Ply*-N genes with cycle thresholds of 33 and 34, respectively. POC real-time PCR detection of enterovirus, herpesvirus, varicella-zoster virus and *Neisseria meningitidis* remained negative in the CSF [5]. CSF grew colonies on chocolate agar and 5 % sheep-blood agar (bioMérieux, Marcy l’Etoile, France) after a 5-day incubation period at 37 °C under a 5 % CO_2_ atmosphere. Colonies were identified as *S. pyogenes* by matrix-assisted laser desorption-ionization time-of-flight mass spectrometry (MALDI-TOF-MS) with an identification score of 2.26 [7]. Antibiotic susceptibility testing using the disk diffusion method found the *S. pyogenes* isolate to be susceptible (according to EUCAST guidelines) to amoxicillin [minimum inhibitory concentration (MIC), 0.250 mg/L], ceftriaxone (MIC, 0.5 mg/L), rifampicin (MIC, 0.052 mg/L), clindamycin (MIC, 0.4 mg/L) and doxycycline (MIC, 4 mg/L), but resistant to erythromycin (MIC, 1 mg/L). *S. pneumoniae* was not cultured from the CSF, though both *S. pneumoniae* and *S. pyogenes* were cultured from the ear pus after 1-day and 2-day incubations at 37 °C and 5 % CO_2_, respectively. Colonies were identified by MALDI-TOF-MS with identification scores of 2.22 and 2.35, respectively. The ear pus *S. pyogenes* isolate exhibited the same antibiotic susceptibility pattern as the CSF *S. pyogenes* isolate. The antibiotic susceptibility of the ear pus *S. pneumoniae* isolate, tested by using the E-test method (BioMérieux), indicated *in vitro* susceptibility to penicillin (MIC, 0.012 mg/L), amoxicillin (0.016 mg/L), ceftriaxone (0.016 mg/L), imipenem (0.004 mg/L) and vancomycin (0.250 mg/L). Susceptibility testing to oxacillin, gentamicin, erythromycin, rifampicin, clindamycin and doxycycline by using the disk diffusion method found the isolate to be susceptible to all these antibiotics.

One day after admission, our patient suffered epileptic seizures resistant to 1 mg clonazepam. An electroencephalogram confirmed status epilepticus and our patient was given sodium valproate and levetiracetam and was admitted to the intensive care unit. Cefotaxime (18 gr/day) was intravenously administered with a syringe pump for 14 days in association with dexamethasone the first day. Our patient eventually recovered after 4 weeks of hospitalization. A follow-up at 4 months postdischarge found no sequelae. The otolaryngologist prescribed long-term treatment with amoxicillin to prevent any further otitis.

## Conclusions

In this patient, mixed meningitis due to both *S. pneumoniae* and *S. pyogenes* was not initially suspected after direct microscopic examination of the CSF disclosed Gram-positive cocci*.* Indeed, although subtle microscopic differences have been reported that distinguish both streptococci, they are unreliable for prospectively distinguishing between both pathogens in routine practice. Detection of *S. pneumoniae* antigen by using immunochromatographic tests and real-time PCR, however, enables rapid detection of *S. pneumoniae* in the CSF at the point of care [5].

Moreover, the case here reported of a mixed infection is exceptional as no such case has ever been previously reported. Indeed, while *S. pneumoniae* is the leading bacterial etiology of purulent meningitis in adults [1], *S. pyogenes* meningitis is by itself a rare condition, and is reported in a total of 29 adult patients, including the one reported here [8–11]. *S. pyogenes* meningitis has a poor prognosis; six patients died and most of the other patients showed neurological complications (Table 1). That was not the case for the patient reported here.

**Table 1:** Review of main characteristics of *Streptococcus pyogenes* meningitis cases in the literature

Characteristic of the 29 patients	
Age (median, years)	45
Female	17 (59 %)
CSF examination	
Leukocyte count	1198/mm^3^
Protein	2.3 g/L
Predisposing conditions	
Otitis	23 (79 %)
Sinusitis	7 (24 %)
Outcome	
Acute neurological complications	19 (66 %)
Neurologic sequelae	11 (38 %)
Hearing loss	4 (14 %)
Death	6 (21 %)

*CSF* cerebrospinal fluid

In our patient, purulent otitis was confirmed as the portal of entry by culture of both *S. pneumoniae* and *S. pyogenes* exhibiting the same antibiotic susceptibility pattern as the CSF isolate. We hypothesized that in this patient with purulent otitis, a 12-hour flight led to a barotraumatic breach of the tympanus and central nervous system infection. Barotrauma following flight has been previously reported [12]. Moreover, one similar case of *S. pneumoniae* meningitis following pneumococcal otitis after scuba diving has been described [13]. Patients diagnosed with chronic lymphocytic leukemia, as was the case in the patient here reported, are at increased risk of systemic infection by encapsulated bacteria such as *S. pneumoniae*. In such patients, pancytopenia at the time of admission is an additional risk factor [14].

In the patient reported here, both streptococci tested susceptible to cefotaxime, the empirical treatment administered to our patient and successfully continued for 14 days. This case indicates that immunocompromised patients should avoid barotrauma when presenting with acute uncontrolled bacterial otitis. Concerning their sensitivity to pneumococcal infections, patients should be offered the 13-valent pneumococcal conjugate vaccine at the time of their chronic lymphoid leukemia diagnosis [15].

## Consent

Written informed consent was obtained from the patient for publication of this case report and any accompanying images. A copy of the written consent is available for review by the Editor-in-Chief of this journal.
